# Automatic Diagnosis of Pathological Myopia from Heterogeneous Biomedical Data

**DOI:** 10.1371/journal.pone.0065736

**Published:** 2013-06-14

**Authors:** Zhuo Zhang, Yanwu Xu, Jiang Liu, Damon Wing Kee Wong, Chee Keong Kwoh, Seang-Mei Saw, Tien Yin Wong

**Affiliations:** 1 Institute for Infocomm Research, Agency for Science, Technology and Research, Singapore, Singapore; 2 School of Computer Engineering, Nanyang Technological University, Singapore, Singapore; 3 Saw Swee Hock School of Public Health, National University of Singapore, Singapore, Singapore; 4 Department of Ophthalmology, Singapore Eye Research Institute, Singapore, Singapore; Vanderbilt University, United States of America

## Abstract

Pathological myopia is one of the leading causes of blindness worldwide. The condition is particularly prevalent in Asia. Unlike myopia, pathological myopia is accompanied by degenerative changes in the retina, which if left untreated can lead to irrecoverable vision loss. The accurate diagnosis of pathological myopia will enable timely intervention and facilitate better disease management to slow down the progression of the disease. Current methods of assessment typically consider only one type of data, such as that from retinal imaging. However, different kinds of data, including that of genetic, demographic and clinical information, may contain different and independent information, which can provide different perspectives on the visually observable, genetic or environmental mechanisms for the disease. The combination of these potentially complementary pieces of information can enhance the understanding of the disease, providing a holistic appreciation of the multiple risks factors as well as improving the detection outcomes. In this study, we propose a computer-aided diagnosis framework for Pathological Myopia diagnosis through Biomedical and Image Informatics(PM-BMII). Through the use of multiple kernel learning (MKL) methods, PM-BMII intelligently fuses heterogeneous biomedical information to improve the accuracy of disease diagnosis. Data from 2,258 subjects of a population-based study, in which demographic and clinical information, retinal fundus imaging data and genotyping data were collected, are used to evaluate the proposed framework. The experimental results show that PM-BMII achieves an AUC of 0.888, outperforming the detection results from the use of demographic and clinical information 0.607 (increase 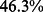, 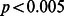), genotyping data 0.774 (increase 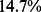, 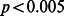) or imaging data 0.852 (increase 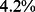, 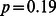) alone. The accuracy of the results obtained demonstrates the feasibility of using heterogeneous data for improved disease diagnosis through our proposed PM-BMII framework.

## Introduction

### Pathological Myopia

Pathological myopia (PM) is one of the leading causes of visual impairment worldwide[1]–[3] and is the most frequent cause of visual impairment in Asian countries [4]. Known as high myopia or degenerative myopia, pathological myopia is a type of severe and progressive nearsightedness characterized by changes in the fundus of the eye, due to posterior staphyloma and deficient corrected acuity. It is commonly defined as having a spherical equivalent (SE) of at least −6.0 diopters [5]. Pathological myopia causes very rapid changes in vision, often requiring a change in eyeglasses or contact lens prescriptions every 4 to 6 months. This condition usually does not stabilize within normal limits, thus affecting the curvature of the crystalline lens and increasing the risk of retinal detachment. Myopia-related visual impairment has been shown to affect productivity and quality of life. As patients with pathological myopia are more prone to ocular abnormalities, it is increasingly essential to manage the progression of degenerative myopia with early detection and treatment. Current clinical practice in detecting pathological myopia relies heavily on the manual screening and efforts of the clinicians, where a complete eye exam usually takes up to 60 minutes. Such eye exams include questions on the subject’s medical history and a physical eye examination which includes tests for visual acuity, visual field and refraction. For example, a slit lamp exam evaluates the anterior sections and lens of the eye using microscope optics; tonometry measures the pressure inside the eye; and ophthalmoscopy allows observation of the back of the eye.

### Pathological Myopia Diagnosis via Learning from Heterogeneous Biomedical Data

Recently, there has been increasing interest in the development of retinal imaging algorithms and computer-aided diagnosis (CAD) systems to automatically detect pathological myopia from retinal fundus images towards screening. For example, Liu *et. al.* [6] presented the PAMELA system to detect pathological myopia in fundus images through the detection of parapapillary atrophy (PPA) around the optic disc. Zhang *et. al.* [7] combined fundus image data and demographic/clinical data to identify an optimal set of essential features to improve the prediction of pathological myopia. Moreover, with genotyping technologies out-pacing Moore’s Law since 2008 [8], it has become much less costly to obtain genomic information, in particular SNP (Single Nucleic Polymorphism) data. SNP data provides partial view of a person’s genetic profile, and the known disease associated SNPs can be used as a form of genetic prior knowledge in gauging the likelihood of disease occurrence.

Each of these heterogeneous data sources (fundus, demographic/clinical, genetic) is likely to contain a different perspective on the disease risk of an individual, based on the pathological, environmental and genetic mechanisms of the disease. These perspectives may potentially be complementary, such that a combination of the data from these independent sources are able to provide a more comprehensive and holistic assessment of the disease [9]. Furthermore, data from these sources are becoming increasingly available. Retinal fundus imaging can be found in numerous primary community healthcare institutions as well as optical shops. With the dramatic reduction in genotyping costs in recent years, it is foreseeable that SNP data can be acquired at low cost and with as ease as demographic clinical data in the near future. The objective of our study is to develop a computational tool in facilitating automatic predictions for applications such as health screening when clinicians are not present but abundant data is available.

In this work, we propose a computer-aided framework for the detection of pathological myopia called PM-BMII (Pathological Myopia diagnosis through Biomedical Image Informatics). The PM-BMII framework uses a data-driven approach to exploit the growth of heterogeneous data sources to improve assessment outcomes. One challenge in this approach is the disparity of labels used to describe such data. For example, imaging data is represented by an image, while demographic/clinical data is described by quantitative measurements or categorical data and SNPs are coded by text representing the nucleotide combinations. To address this challenge and combine such data meaningfully, a SVM-based multiple kernel learning algorithm is proposed in our PM-BMII framework.

### SVM Based Multiple Kernel Learning

Over the past twenty years, Support Vector Machines (SVM) [10], [11] have become a ubiquitous tool in machine learning. SVM algorithms distinguish themselves from other margin-maximizer classifiers through the use of kernel functions, which transform the input data before classification. In traditional SVM algorithms, a single kernel function is applied on all input data. While convenient and efficient for homogeneous data, the use of a single kernel can result in compromises in performance when used in models combining heterogeneous data type.

Recent extensions to the SVM framework have described the use of multiple kernels. Such approaches, commonly known as Multiple Kernel Learning (MKL) algorithms [12], [13], allow us to combine heterogeneous feature sets, each with their own adapted kernel function, while optimizing the contribution of each sub-kernel to the resulting classifier. Furthermore, in a standard single kernel SVM, it is difficult to determine the importance of an individual feature. The advantage of MKL is such that it generates weights for each sub-kernel which can provide a useful representation of the relative discriminative power of each set of features.

## Materials and Methods

### Proposed Framework PM-BMII

**Figure 1:**
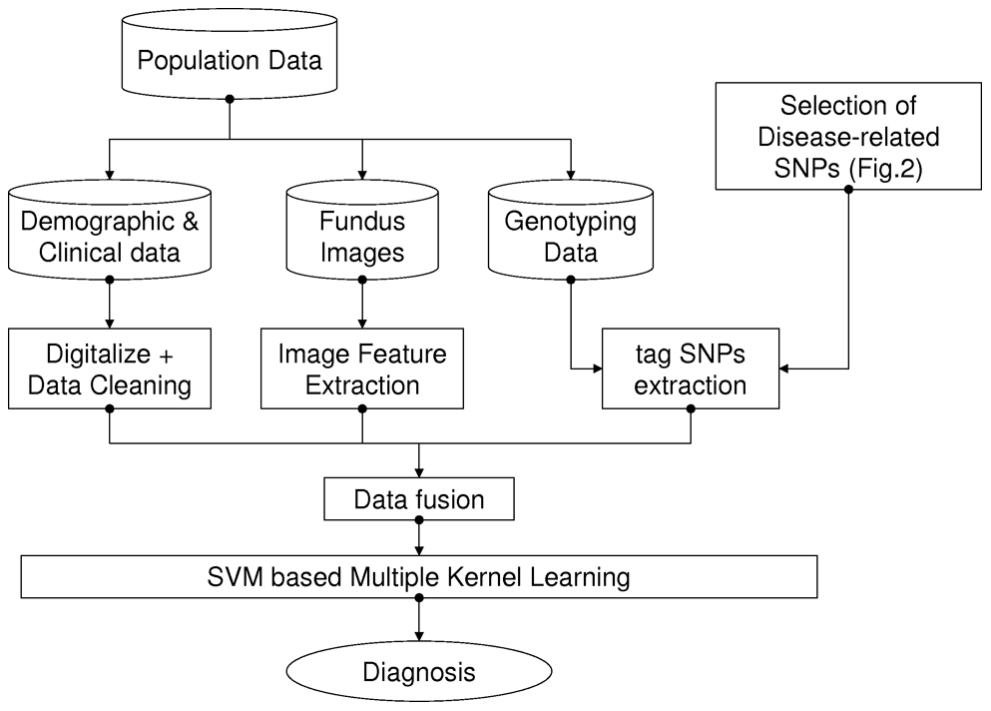
Architecture of PM-BMII framework.

In this work, we propose a computer-aided diagnosis framework for the detection of pathological myopia called PM-BMII. The framework automatically detects pathological myopia based on a combination of heterogeneous sources, i.e. imaging data, demographic/clinical data, and genotyping data. We use an MKL-based approach to optimize modeling, learning and classification. Figure 1 illustrates the architecture of the proposed PM-BMII framework.

### SiMES Data Description

We evaluate the proposed PM-BMII framework on the Singapore Malay Eye Study (SiMES) database [14]. SiMES examined a population-based, cross-sectional, age stratified, random sample of 3280 Malays (78.7% participation rate) aged 40 to 80 years living in Singapore. A subject’s demographic variables, fundus photograph and blood sample for genotyping were acquired during the clinic visit. The diagnosis of pathological myopia was made at the same time. We use the clinical diagnosis of PM as the gold standard to evaluate our approach.

In current clinical settings, fundus images are easily available at polyclinics and even optical shops. Furthermore, the cost of genotyping chips has decreased dramatically in recent years, a trend of which would greatly increase the accessibility of a person’s genotyping data in the near future. The objective of our study is to develop a computational tool facilitating automatic prediction for applications such as health screening when clinicians are not present but abundant data is available.

The following data is used to evaluate the proposed PM-BMII framework:

- Fundus Image Data: The images were acquired using a 45° FOV Canon CR-DGi retinal fundus camera with a 10D SLR backing, at an image resolution of 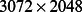 pixels
- Demographic/clinical Data: The eye screening record in SiMES contains demographic/clinical data such as age and gender, medical histories (e.g. diabetes etc.) and ocular examination data. The clinical diagnosis of pathological myopia is used as the gold standard label in this study.
- Genotyping (SNP) Data: subjects were genotyped on Illumina 610quad arrays, followed by a stringent quality control (QC) procedure [15]. The QC process excludes the subjects with a missing call rate 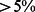, filters out monomorphic SNPs, non-autosomal SNP and SNPs with minor allele frequency (MAF) 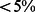. A Hardy-Weinberg equilibrium (HWE) test was also conducted to detect genotyping artifacts [16]. The final SNP data set contains 2,542 individuals with 557,824 SNPs on 22 autosomal chromosomes.

### Knowledge-based Feature Selection in SNP Data

It has been shown that there is an interplay between genetic factors and environmental influences [17] in myopia, with an estimated heritability of myopia at 0.306 [18]. A set of myopia related genes were discovered in linkage studies [19]–[21], and recent genome wide association studies (GWAS) further identified several loci highly associated with pathological myopia [22]–[26]. This valuable knowledge forms a *smart prior* in our framework, and using such a *smart prior* for feature selection enables us to overcome the *curse of dimensionality* raised by the overwhelming number of SNPs as compared to samples. We propose a holistic approach to identify myopia-related SNPs using the following steps:

- Identify susceptibility loci from a group of myopia-related genes
- We use the OMIM (Online Mendelian Inheritance in Man) database [27] to obtain disease related SNPs. OMIM contains information on known genetic disorders and over 12,000 genes, with carefully examined reference literature. We searched OMIM with query item *myopia [TI]* and found 40 entries, from which a list of myopia-related SNPs were extracted as shown in table 1.
- Obtain susceptibility loci from recent published genome wide association study
- We used the NHGRI GWAS catalog [28] to search for PM-associated SNPs discovered by recent Genome Wide Association Studies [22]–[26]. The SNPs and their references are listed in Table 2.
- Match tag SNPs genotyped in SiMES data
- The SNPs identified in the above steps may not appear as markers genotyped in SiMES data. Based on the fact that Illumina 610quad arrays are derived from the International HapMap Project [29] with one tag SNP every 5–6 kb across the genome in the CEU,CHB+JPT and YRI populations, we use GVS (Genome Variation Server) [30] to find corresponding tag SNPs. The GVS database contains 11.8 million SNPs with corresponding genotyping data and provides a set of tools for the analysis of SNP data. For each SNP identified in Steps 1 and 2, we set a range of 3 kb both up- and down-stream with a LD-score 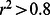 as the search criteria to catch the corresponding tag SNPs.

**Table 1:** Pathological Myopia (PM) related SNPs found from Genetic Linkage Studies.

Genes	Location	OMIM ID	PM SNP	Source
MYP2	18p11.31	160700	rs1034762, rs1635529, rs1793933, rs3803183, rs17122571	Young, Ronan, Drahozal et al. (1998), Mutti et al. (2007), Metlapally et al. (2009)
MYP3	12q21-q23	603221	rs3832846, rs17853500, rs3759223, rs10860860, rs2946834, rs6214	Young, Ronan, Alvear et al. (1998), Lin et al. (2010), Metlapally et al. (2010)
MYP7	11p13	609256	rs1506, rs592859, rs608293, rs628224, rs662702, rs667773, rs694617, rs1540320, rs1806155, rs1806158, rs1806159, rs1806180, rs1894620, rs2071754, rs2239789, rs3026389, rs3026401	Hammond et al.(2004)
MYP11	4q22-q27	609994	rs113432966, rs112669274, rs112391551, rs112356377, rs111691784, rs111322719	Zhang, Guo et al. (2005)
MYP12	2q37.1	609995	rs111706042	Paluru et al. 2005
MYP13	Xq23-q25	300613	rs113695792, rs111774596	Zhang, Guo et al. 2006
MYP14	1p36	610320	rs113328794	Stambolian et al. (2004)
TGIF	18p11.31	602630	rs121909066, rs121909067, rs121909068, rs121909069, rs121909070, rs28939693	Gripp et al. (2000)

**Table 2:** Pathological Myopia (PM) associated SNPs found in Genome-wide Association Studies (GWAS).

Genes	Location	PM SNP	Source
GJD2	15q14	rs634990	Solouki et al. 2010, Nature Genet.
RASGRF1	15q25	rs939661	Hysi et al. 2010, Nature Genet.
CTNND2	5q15	rs6885224, rs12716080	Li et al. 2011, Ophthalmology
MIPEP	13q12.12	rs9318086	Shi et al. 2011b, AJHG
ZC3H11B	1q41	rs4373767	Fan et al. 2012, PloS Genetics
LAMA2	6q22.33	rs12193446	
CD55	1q32.2	rs1572275	
ZNF644	1p22.2	rs6680123	Shi et al. 2011a, Plos Genetics
MYP11	4q25	rs10034228, rs1585471	Li et al. 2011, Hum Mol Genet.
BLID	11q24.1	rs577948	Nakanishi et al. 2009, Plos Genetics
GLULP3		rs12275397	

**Figure 2:**
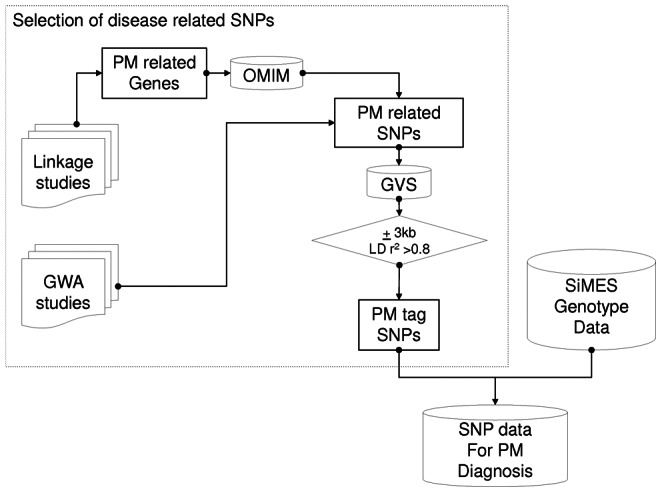
Knowledge-based SNP selection in genotyping data.

Figure 2 illustrates the steps described above. A detailed list of the extracted SNPs are listed in Table 1 and 2. In total 87 SNPs are matched in SiMES genotyping data and these SNPs are used to form a sub-feature space for learning.

### Demographic and Clinical Data Preprocessing

Both environmental and genetic factors have been associated with the onset and progression of myopia. Some of the known environmental risk factors of myopia include *close up work, educational level, IQ, outdoor activity, academic achievement* and *an introvert personality* [17]. These risk factors are partially represented in the demographic and clinical variables obtained from the population study protocol. The data is cleaned by removing subjects or variables with more than 5% missing values. We digitized the categorical parameters and scaled all variables to range of [0,1]. The clean set contains 44 parameters as listed in Table 3, with 2,258 subjects data matched with image and SNP data.

**Table 3:** List of Demographic & clinical variables used in PM-BMII.

Age	Blood LDL Cholesterol	Can read
Age Group	Blood HDL Cholesterol	Can write
Gender	Triglycerides	Alcoholic drink categories
Height	Hypertension	Ever Smoke
Weight	Hypertension treatment & control	Current smoker
Diastolic Blood Pressure	Albumin-Creatinine ratio	Angina
Systolic Blood Pressure	Diabetes I	Heart Attack
Pulse Pressure	Diabetes II	Stroke
Mean arterial pressure	Job Categories	Hypercholessterolemia
BMI	Race	Thyroid Condition
Blood Creatinine	Marital Categories	Chronic Kidney Disease indicator
Blood Glucose	Income Categories	hyperlipidemia
Blood HbA1c Categories	Type of place living in	Metabolic syndrome
Blood Glycosylated Haemoglobin	Place of birth	Microalbuminuria
Blood Total Cholesterol	Education categories	

We conducted a univariant analysis for all parameters. P-values are obtained by conducting the Student’s T-test for numerical variables and the Chi-square test for categorical variables. The following parameters were found to be associated with pathological myopia with P-value 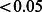 : *Age* (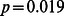), *Job Category* (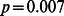), *Income* (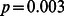), *Type of place living in* (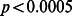), *Education* (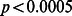), *Ever Smoke* and *Current Smoke* (both 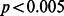).

### Semantic Image Feature Analysis for Fundus Image

Semantic image features, also known as high-level features, differ from low-level local features as they are global features which are location-independent. In this work, the bag-of-words (BOW) model approach from computer vision [31] is introduced for semantic image feature extraction.

**Figure 3:**
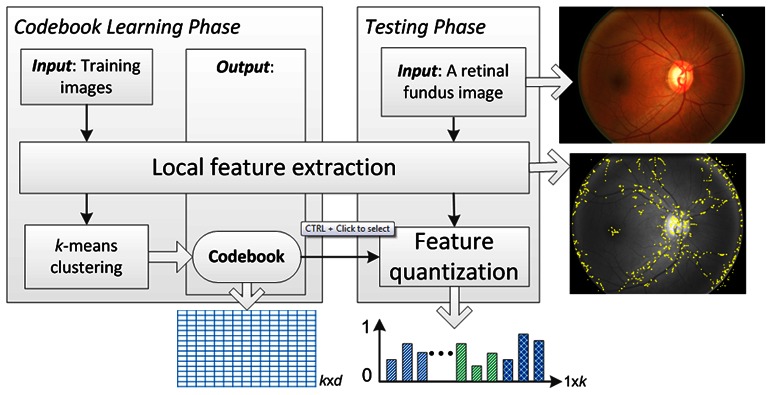
Semantic image feature extraction.

BOW is a simplified representation used in natural language processing and information retrieval by treating local image features as words. In natural language processing, a bag-of-words is a sparse vector of occurrence counts of words; that is, a sparse histogram over the vocabulary. Correspondingly, in computer vision, a bag-of-words is a sparse vector of occurrence counts of a vocabulary of local image features (codebook), which is a location-independent global feature. The properties of local features, such as intensity, rotation, scale and affine invariants can also be preserved. Figure 3 illustrates the described method.

Many visual features can be extracted from grids or superpixels [32] to form local features, such as histogram of oriented gradients (HOG) [33], biologically inspired features (BIF) [34] and color histograms [35] which are related to edges, textures and intensity, respectively. In this work, SIFT (Scale-invariant feature transform) [36] features are used as local features. SIFT has been widely used in object detection and classification, due to its intensity, rotation, scale and affine invariant properties. In this implementation, the Harris-Laplacian (HAR) and Hessian-Laplacian (HES) detectors [37] were used to generate SIFT features from each retinal fundus image. This is mainly because both detectors produce complementary features: HAR locates corner features, while HES extract blob features. Each SIFT feature was represented as a 128-dimensional histogram and each dimension was quantized into an integer between 0 and 255.

To reduce computational costs and avoid feature noise from the retinal image field of view limits, the images were resized to a height of 256 pixels by keeping the original aspect ratio, and only feature points within 0.95 radius to the center were collected for further processing. In addition, the SIFT feature extraction was performed on the green channel only, since the retinal images are less well differentiated in the red and blue channels.

After obtaining all the SIFT features from training images, k-means clustering was used to generate the codebook by randomly selecting half of the training images, with each cluster centroid representing a visual word. After which the BOW global features (*i.e.*, occurrence counts of the visual words in a retinal image) of each training and testing image were obtained in the quantization procedure. To balance the dimensions of different features, we empirically set k = 100. 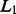-normalization is performed to standardize features before training and testing.

### Data Fusion

The features extracted from each of the three heterogeneous data sets were merged via subject matching. The final dataset contains 2,258 subjects with demographic/clinical data, fundus image and SNP data. Among the 2,258 individuals, 58 had been diagnosed with pathological myopia while the rest were normal. The distribution of pathological myopia subjects in the dataset is representative of the prevalence of pathological myopia in the population. The range of each feature dimension was normalized to the range of [0, 1] in order to avoid magnitude differences among the dimensions.

### Learning Algorithms

In this study, we apply SVM-based multiple kernel learning (MKL) to train the classifier in the proposed PM-BMII framework. The learning problem can be formulated as follows. Given a training set of instance-label pairs 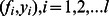 where 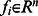 represents the features of a subject, and 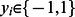 denotes its label, such that 1 denotes the presence of pathological myopia, and −1 denotes the absence of the disease, the basic SVM [10], [11] formulation requires the solution of the following optimization problem:
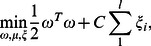
(1)subject to 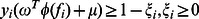
where a feature 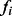 is mapped into a higher dimensional space by the function 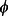. SVM finds a linear separating hyperplane with the maximal margin in this higher dimensional space. 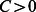 is the penalty parameter of the error term. 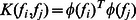 is called the kernel function.

In our experiments, when only one type of feature set (e.g., SNP) is used, a linear kernel 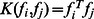 based basic SVM classifier is utilized, where the corresponding label of 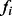 is determined by 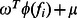.

When incorporating feature sets from multiple data sources, multiple kernel learning (MKL) is applied to learn the adapted kernel function for each feature set, and to optimize the contribution of each sub-kernel for the resulting classifier. In such cases, a convenient approach is to consider that 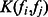 is actually a convex combination of the basis kernels:
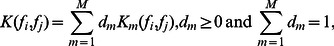
(2)where 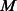 is the total number of kernels. Each basis kernel 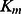 may either use the full set of features describing samples or subsets of features stemming from different data sources [12]. Within this MKL framework, the problem of data representation through the kernel is then transferred to the selection of weights 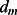. In PM-BMII, we use basis kernels based on each single data source, and demonstrate that models based on a combination of multiple sources are better than those using a single data source. For efficiency, one linear kernel is initialized for each feature type. There are many MKL solver toolboxes which are publicly available, such as SimpleMKL [38] and Group Lasso [39]. The LIBLINEAR toolbox [40] is used to train linear SVM models for each individual data source, and the Group Lasso [39] toolbox is used to train MKL models.

### Experimental Methods for PM-BMII

To demonstrate that the combination of multiple data sources can enhance detection accuracy in our PM-BMII framework, we report and compare the diagnosis performance of 7 methods using the following different features and their combinations:

1. Demographic/clinical data only (referred to as 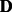)
2. SNP data, genetic information only (referred to as 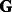)
3. low-level direct image features only (referred to as 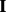)
4. combined demographic/clinical data and SNP data (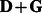)
5. combined demographic/clinical data and image features (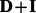)
6. combined SNP data and image features (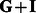)
7. combined all three data source, 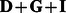 (PM-BMII)

For fair comparison, we performed 10 independent tests, with two rounds of stratified cross-validation conducted per test. This was carried out in the following way. In each test, all subjects were randomly divided into non-overlapping sets of equal size, A and B. In the first round, we used all the positive subjects and the same number of randomly selected negative subjects from set A as training set, due to the imbalanced in the number of positive (PM) and negative (normal) subjects. The trained model is then used for testing set B. The second round was conducted in the same approach but with subjects from set B used for training and those from set A used for testing. In total, 20 groups of evaluation results were collected for each of the 7 methods for analysis.

### Analysis Methods used for PM-BMII

We assess the classification performance using the area under the ROC (receiver operating characteristic) curve (AUC) *which evaluates the overall performance* and a balanced accuracy with a fixed 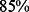 specificity. The balanced accuracy (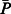), sensitivity (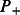) and specificity (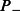) are defined as
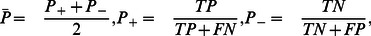
(3)where 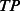 and 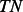 denote the number of true positives and negatives, respectively, and 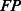 and 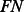 denote the number of false positives and negatives, respectively.

## Results and Discussion

Table 4 shows the results for the different input data, both single and combined, on their ability to detect pathological myopia, measured using the specificity, sensitivity and area under the ROC curve (AUC). The mean and standard variation (SD) values of AUC of each method were calculated based on the results obtained from the 20 sets of cross validation testing as described in the above Methods section. At the screening-based specificity setpoint of 0.85, in comparing only the models from single sources, the results show that the use of imaging data provided the best prediction of pathological myopia (Sensitivity 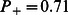), compared to that of SNP data (Sensitivity 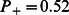) and showed a large improvement over detection using only demographic data (Sensitivity 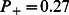). Comparatively, detection using only demographic/clinical data was the least accurate compared to the other single sources.

**Table 4:** Sensitivity and AUC results for the various sources combinations.

source	combination type	sensitivity (specificity = 0.85)	AUC mean	AUC SD
SNP(G)	Single	0.52	0.774	0.038
retinal image(I)		0.71	0.852	0.044
demographic/clinical(D)		0.27	0.607	0.044
G+I	Two	0.73	0.875	0.032
D+G		0.56	0.792	0.037
D+I		0.71	0.863	0.033
D+G+I	Multiple	**0.77**	**0.888**	**0.032**

Results show PM-BMII is better able to detect pathological myopia compared to the other individual or combined sources.

Notes:

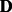
 Demographic/clinical data; 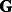 SNP data, genetic information; 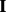 low-level direct image features.

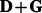
 combined demographic/clinical data and SNP data.

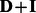
 combined demographic/clinical data and image features.

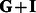
 combined SNP data and image features.

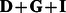
 combined all three data source -(PM-BMII).

When multiple (2 or more) data sources are combined, the general trend shows that the sensitivities from combining sources outperforms their component sources at specificity of 0.85, with the best performing model based on the combination of SNP data, demographic/clinical data and imaging data in the PM-BMII framework (Sensitivity 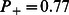).

**Figure 4:**
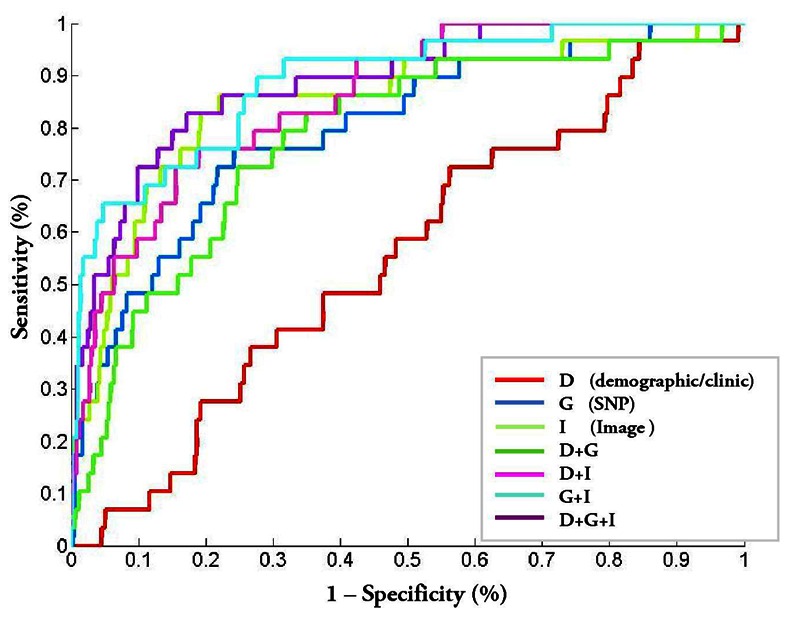
ROC (receiver operating characteristic) curve of various methods.

**Figure 5:**
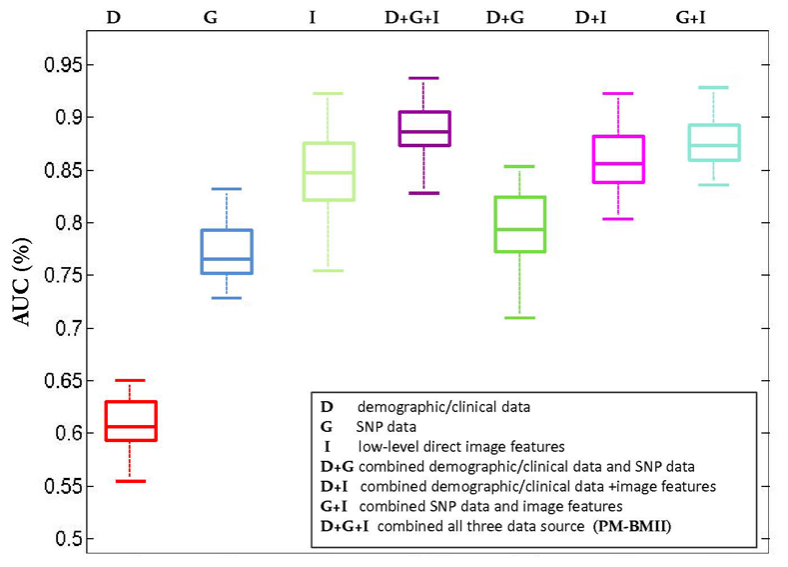
Boxplot of AUC to compare various methods.

This trend can also be observed using the calculated AUC metrics, with the corresponding ROC plots presented in Figure 4 and box plot of AUC distribution based on the 20 rounds of cross validation tests shown in Figure 5. The PM-BMII prediction model combining demographic/clinical data, SNP data and imaging data generated the best AUC metrics (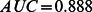), outperforms all other source combinations or single sources. Compared to the single sources, the use of PM-BMII resulted in significant improvements over demographic/clinical data 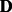 (increase 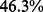, 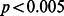) and genetic information 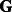 (increase 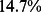, 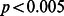). It also tended toward better classification than using imaging data 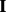 alone (increase 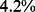, 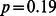). Furthermore, the results also show PM-BMII performs better than the combined models obtained from the combinations of any two sources, resulting in improvements of 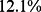 (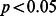), 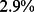 (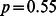) and 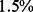 (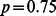) in AUC over 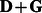, 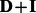 and 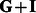 respectively.

Our experimental results also suggest an advantage in combining any two sources over the use of their component sources. For example, the use of SNP and retinal image information 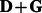 produced an AUC of 0.792, which is better than the individual AUCs from 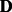 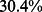 (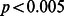) and 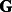 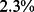 (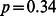) respectively. This trend can also be observed for the other two combinations 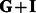 and 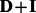 over their components.

In this work, we have tested the use of different combinations of data for the detection of pathological myopia. These data sources can be described as imaging data, SNP data and demographic/clinical data. Based on the results of the experiments, the following observations can be made:

1. PM-BMII approach of combining imaging, SNP and demographic/clinical data outperformed single data sources and two-source combinations
2. In our experiments, we have shown that the combination of imaging, genomic and demographic data in the PM-BMII framework was able to achieve an AUC of up to 0.888. The PM-BMII prediction results outperform the models based on other data combinations, as well as the individual component sources.
3. Advantages in combining different data types
4. Furthermore, the experiments also support combining different data types for pathological myopia prediction. In the experiments based on the combinations of any two different types, we observed that the results were better than the models which only use the individual data components. This was most obvious in the use of demographic/clinical data 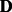, which when used in conjunction with any other data type registered an improvement of at least 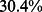 (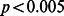) in pathological myopia detection. Although the use of individual data can possibly be used for detection, our results show that it is advantageous to include at least one other data type in the model. This suggests that the data types are indeed complementary.
5. Usefulness of demographic/clinical data
6. The results show that the performance of PM-BMII (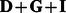) is comparable to that of using SNP and imaging information 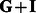. However, the addition of demographic/clinical data 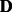 to genetic information 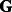 or 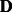 to 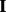 does show a trend of improving accuracy. This suggests that in the overall PM-BMII framework the inclusion of demographic/clinical data 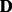 may not be strictly necessary, particularly when both genetic information 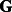 and imaging information 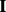 are included, and further suggests some possible redundancy in the use of demographic/clinical data 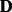 with genetic and imaging information 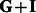. Nonetheless, a model that is built using imaging information I or SNP data 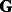 alone would benefit from the inclusion of demographic/clinical data 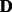.
7. We observe the limited significance of adding SNP and demographics data into the prediction model, with a modest 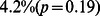 improvement of AUC. This may be due to the limited number of subjects in our study. Increasing data available in future studies could allow us to draw more significant conclusions.

### Conclusions

Demographic/clinical data, imaging data and SNP data can provide different perspectives towards disease detection. With the large quantity of potential data that can be obtained, the challenge is to combine these data in a holistic fashion to make the best use of their individual advantages. Computer-based informatics methodologies offer such an opportunity to intelligently fuse these data sources. We have proposed PM-BMII, a framework powered by MKL, for Pathological myopia diagnosis by combining heterogeneous biomedical data, including demographic data, imaging data and SNP data. Our experiments show that the PM-BMII framework is able to detect pathological myopia with high accuracy, and supports the use of data fusion over any single or two-source combination. These promising results encourage further exploration of the PM-BMII framework for the detection of other ocular diseases.
